# Effects and safety of massage therapy for patients with metatarsal pain: A protocol for systematic review and meta-analysis

**DOI:** 10.1097/MD.0000000000023484

**Published:** 2020-12-04

**Authors:** Ke-Lin Zhou, Shuo Dong, Xiao Bai, Xiao-Hui Dai, Sheng Guo, Qing Guan, Pei-Dong Wei, Bao-Lai Mi, Mei-Ling Ren, Yong Yang

**Affiliations:** a Dongfang Hospital of Beijing University of Chinese Medicine; b School of Traditional Chinese Medicine, Beijing University of Chinese Medicine, Beijing, China.

**Keywords:** complementary and alternative medicine, massage therapy, metatarsal pain, metatarsalgia, protocol

## Abstract

**Background:**

Metatarsalgia refers to localized or generalized forefoot pain in the region of the metatarsal heads. Often this pain is plantar, beneath the metatarsal heads, and arises from either mechanical or iatrogenic causes. The treatment of metatarsalgia remains controversial. A thorough understanding of the biomechanics of the forefoot and the underlying pathology of the particular type of metatarsalgia affecting the patient is a prerequisite to selecting the proper treatment. In recent years, massage therapy has been increasingly accepted by patients due to its lower costs, fewer unwanted side effects, and safety for clinical use. In this systematic review, we aim to evaluate the effectiveness and safety of massage therapy for patients with metatarsal pain.

**Methods:**

We will search the following electronic databases for randomized controlled trials to evaluate the effectiveness and safety of massage therapy in treating metatarsal pain: Wanfang and PubMed Database, CNKI, CENTRAL, CINAHL, and EMBASE. Each database will be searched from inception to October 2020. The entire process will include study selection, data extraction, risk of bias assessment, and meta-analyses.

**Results:**

This proposed study will evaluate the effectiveness and safety of massage therapy for patients with metatarsal pain. The outcomes will include changes in metatarsal pain relief and adverse effect.

**Conclusions:**

This proposed systematic review will evaluate the existing evidence on the effectiveness and safety of massage therapy for patients with metatarsalgia.

**Dissemination and ethics:**

The results of this review will be disseminated through peer-reviewed publication. Because all of the data used in this systematic review and meta-analysis has been published, this review does not require ethical approval. Furthermore, all data will be analyzed anonymously during the review process.

**OSF Registration number:**

DOI 10.17605/OSF.IO/C6KFJ

## 1 Introduction

Metatarsalgia manifests itself as a pain in the forefoot, including areas corresponding to the metatarsals and toes.^[1]^ Often this pain is under the distal heads of the metatarsal bones, usually in the second and third rays.^[2]^ Most of the pressure during the toe-off phase of the gait concentrates on this area.^[3]^ Metatarsalgia is a quite common condition, which can lead to swelling and deformity in the forefoot, restricting movement, or even totally preventing walking.^[4]^ It is one of the most frequent reasons for consultation in podiatry, especially among athletes and women.^[5]^ A fundamental etiological component of metatarsalgia is the repetitive loading of a locally concentrated force in the forefoot during gait. An overload of weightbearing forces may affect the entire forefoot or an isolated area (e.g., a metatarsal head) when the foot is plantigrade. Metatarsalgia arises from structural deformities such as pes planus, pes cavus, abnormal subtalar joint pronation, or dorsiflexed first ray.^[6]^ In addition, possible causes may associated with acquired foot deformities due to the loss of cushioning capacity in the soft tissue.^[7,8]^ These deformities may come from the individual's increasing age, certain metabolic diseases such as diabetes, or from the repetitive impacts made by the feet in the individual's physical activity.^[9]^

The treatment of central metatarsalgia should address the underlying pathology, as well as the expectations, of the patient. Conservative treatment should always be maximized before considering surgery in the management of this condition.^[10]^ Conservative treatment may include stretching exercises, shoe modification, shaving of the callosity, rest, use of metatarsal pads and molded insoles, corticosteroid injections, and anti-inflammatory medications.^[11,12]^ If nonoperative treatment is unsuccessful, surgical treatment may be warranted.^[13]^ The primary goal of surgery is to restore a normal distribution of pressure within the forefoot. As a rule, it is important to restore a harmonic Maestro curve, to restore the correct metatarsal slope, and to provide adequate ground contact for the metatarsal heads.^[14]^ A thorough understanding of the biomechanics of the forefoot and the underlying pathology of the particular type of metatarsalgia affecting the patient is a prerequisite to selecting the proper treatment. Massage therapy, one of the most popular complementary and alternative therapies, have been used for thousands of years in China. Currently, they are increasingly used because of their lower costs and safety for clinical use.^[15]^

This review aims to systematically review all randomized controlled trials (RCTs) to assess the effectiveness and safety of massage treatment for patients with metatarsal pain.

## 2 Materials and methods

This systematic review protocol has been registered on OSF on October 23, 2020 (Registration number: DOI 10.17605/OSF.IO/C6KFJ). The protocol follows the Cochrane Handbook for Systematic Reviews of Interventions and the Preferred Reporting Items for Systematic Reviews and Meta-Analysis Protocol (PRISMA-P) statement guidelines.^[16]^ We will describe the changes in our full review if needed.

## 3 Inclusion criteria for study selection

### 3.1 Type of studies

This review will include clinical RCTs of clinical massage therapy for metatarsalgia patients without any language or publication status restrictions. Non-RCTs, quasi-RCTs, case series, case reports, crossover studies, uncontrolled trials, and laboratory studies will not be included.

### 3.2 Type of participants

Participants who were diagnosed with metatarsalgia according to related guidelines or consensus. All included participants in this review regardless of their age, race, and sex. Pregnant and lactating women will be excluded.

### 3.3 Type of interventions

Interventions will include any type of clinically performed massage for improvement of metatarsalgia. This will include Chinese Massage, Japanese Massage, Thai Massage, Swedish Massage, Tuina, Shiatsu, Remedial Massage, General Massage, Acupressure, Reflexology, Manual Lymphatic Drainage. Studies of metatarsalgia combined with other interventions such as acupuncture, herbal medicines, qigong, and yoga will be considered for exclusion.

Control: no intervention, treatments other than massage (e.g., usual or standard care, placebo, wait-list controls).

### 3.4 Type of outcome measures

#### 3.4.1 Main outcome(s)

The primary outcome at the end of treatment or at maximal follow-up is the clinical effective rate, which is categorized as cure, markedly effective, effective, or ineffective according to clinical symptoms, visual analogue scale score, joint mobility, and daily living ability score, etc.

#### 3.4.2 Additional outcome(s)

The secondary outcomes will include quality of life (the MOS item short from health survey [SF-36]), symptom scores (pain, limited joint mobility, and joint swelling, etc), comparison of therapeutic effects under arthroscopy, and comparison of curative effect of pathological tissue, etc.

## 4 Search methods for the identification of studies

### 4.1 Electronic searches

We will search the following electronic bibliographic databases for relevant trials:

CNKI (China National Knowledge Infrastructure Database, from 1979 to present);
Wanfang Database (from 1990 to present);
PubMed Database (from 2000 to present);
CENTRAL (Cochrane Central Register of Controlled Trials, from 2000 to present);
CINAHL (Cumulative Index of Nursing and Allied Health Literature, from 1937 to present);
EMBASE (Excerpta Medica database, from 1947 to present);
Ovid MEDLINE ALL (Ovid Medical Literature Analysis and Retrieval System Online, from 1946 to present);

In addition, Clinical trial registries, like the Chinese Clinical Trial Registry (ChiCTR), the Netherlands National Trial Register (NTR), and ClinicalTrials.gov, will be searched for ongoing trials with unpublished data.

There will be no language restrictions.

### 4.2 Data collection and analysis

#### 4.2.1 Study identification

We will use EndNote X9 software (Alfasoft Limited, A.W. House, United Kingdom)EndNote X9 software to manage the records of searched electronic databases. The initial selection will involve scanning of the titles and abstracts of the retrieved studies. The full text of relevant studies will then be reviewed for study inclusion, in accordance with the inclusion criteria, by 2 authors (KLZ and SD). Potentially relevant articles will be reviewed independently by 2 authors to determine if they meet the prespecified criteria. Any disagreement between authors will be resolved by consensus with a third author. The study selection procedure will follow and be recorded in the PRISMA flow chart. All the evidence will be assessed by The Grading of Recommendations Assessment, Development and Evaluation (GRADE).

#### 4.2.2 Data extraction and management

According to the inclusion criteria, a standard data collection form will be made before data extraction. The following data will be extracted by 2 authors (KLZ and SD):

General information: Research identification, publication year, the title of the study, first author;
Study methods: study design, sample size, randomization method, allocation concealment, blinding, incomplete report or selecting report, other sources of bias;
Participants: Inclusion and exclusion criteria;
Intervention: motion details, treatment duration, and frequency;
Control: Type of control methods, motion details, treatment duration, and frequency;
Outcomes: Included outcome measures.

#### 4.2.3 Risk of bias assessment

The risk of bias in included studies will be assessed independently by 2 reviewers (KLZ and SD) using the Cochrane Risk of Bias Tool, with any disagreements resolved by consensus or by discussion with a third reviewer. All judgments will be fully described, and the conclusions will be presented in the Risk of Bias figures and will be incorporated into the interpretation of review findings, by means of sensitivity analysis. The risk of bias of each domain will be graded as adequate, unclear, or inadequate. We intend to use the concealment of allocation grading in investigation of any heterogeneity and in sensitivity analysis. Other aspects of study quality including the extent of blinding (if appropriate), losses to follow up, non-compliance, whether the outcome assessment was standardized, and whether an intention to treat analysis was undertaken, will be presented in the risk of bias table describing the included studies and will provide a context for discussing the reliability of the results.

#### 4.2.4 Data analysis

We will use Stata Software [Computer program] (Version 15.1, StataCorp: College Station, TX) to process the meta-analysis. Weighted mean difference (WMD) will be used for continuous variable data, and the combined statistical effects of these 2 are combined. The chi-squared test will be adopted to analyze whether there is heterogeneity in each of the included research questions. *I*^2^ > 50% is a criterion for significant judgment. The fixed effect model is adopted if *I*^2^ ≤ 50%, which is considered to have homogeneity between the studies. The random effect model is adopted if *I*^2^ > 50%, which is considered to have heterogeneity among the studies. The effect size is expressed as 95% confidence interval (CI), and *P* < .05 is considered to be statistically significant.

Sensitivity analyses: Heterogeneity may be due to the presence of ≥1 outlier studies with results that conflict with the rest of the studies. We will perform sensitivity analyses excluding outlier studies. In addition, we plan to perform sensitivity analysis to explore the influence of trial quality on effect estimates. The quality components of methodology include adequacy of generation of allocation sequence, concealment of allocation, and the use of intention-to-treat analysis.

Meta-regression analyses: If data permits, we will perform the meta-regression analyses.

#### 4.2.5 Publication bias

If sufficient number of trials (>10 trials) are found, we will generate funnel plots (effect size against standard error) to investigate publication bias.

#### 4.2.6 Ethics and dissemination

The data used in this systematic review will be collected from published studies. Based on this, the study does not require ethical approval.

## Acknowledgment

The authors would like to thank all the researchers in our working group.

## Author contributions

**Conceptualization:** Kelin Zhou, Shuo Dong, Xiaohui Dai, Qing Guan, Peidong Wei, Baolai Mi, Meiling Ren, Yong Yang.

**Data curation:** Kelin Zhou, Shuo Dong, Xiao Bai, Xiaohui Dai, Baolai Mi, Meiling Ren.

**Formal analysis:** Kelin Zhou, Peidong Wei.

**Funding acquisition**: Yong Yang.

**Methodology:** Shuo Dong.

**Project administration:** Yong Yang.

**Resources:** Kelin Zhou.

**Software:** Shuo Dong, Xiao Bai, Qing Guan.

**Supervision:** Sheng Guo.

**Writing – original draft**: Ke-Lin Zhou, Shuo Dong.

**Writing – review & editing:** Sheng Guo, Yong Yang.
